# HIV/AIDS services quality in health centers of East Shoa zone, Oromia, Ethiopia

**DOI:** 10.4314/ahs.v22i3.48

**Published:** 2022-09

**Authors:** Temesgen Aferu, Gebremedhin Beedemariam, Teferi Gedif

**Affiliations:** 1 School of Pharmacy, College of Health Sciences, Mizan Tepi University, Mizan-Aman, Ethiopia; 2 School of Pharmacy, College of Health Sciences, Addis Ababa University, Addis Ababa, Ethiopia

**Keywords:** HIV/AIDS, Services quality, Health center, East Shoa Zone

## Abstract

**Background:**

HIV/AIDS is a major public health, social and economic problem in Ethiopia. However, little has been done on assessment of the quality of the services given to patients in this country.

**Objective:**

To assess the quality of HIV/AIDS services in health centers of East Shoa Zone, Oromia region, Ethiopia.

**Method:**

Cross sectional survey was undertaken in selected health centers of East Shoa Zone between February and May, 2017. Data was collected using researcher administered structured questionnaire, logistics indicators assessment tool and observation check list. SPSS for windows version 20 was utilized in the analysis of the collected data.

**Results:**

The study facilities were providing various services to HIV/AIDS patients. All (100%) and 6(75%) facilities respectively had shortage of trained human power required to give ART and TB services. Regarding ARV medicines availability, majority of the study facilities, 5 (62.50%) reported that they had the stockout of AZT300/3TC150/NVP200 in six months prior to study while 4 (66.7%) of the facilities had the stockout of NVP 240ml (50mg/5ml) syrup on day of visit. Among anti-TB medicines, E100 was out of stock in three facilities (37.5%) on day of visit and INH100 had been out of stock in 4 (50%) of the facilities in six months prior to the study. From OIs medicines, Cotrimoxazole 960mg tablet stockedout in 4 (66.70%) on day of visit and in 5 (83.30%) health centers in six months prior to the study. Considerable number of study facilities, 4 (66.70%) had the stockout of tramadol 50mg tablet on day of visit and ibuprofen 400mg tablet in six months prior to the study, 5 (71.40%).

**Conclusion:**

The studied facilities were challenged by different factors including, scarcity of human power, stockout of various HIV/AIDS related medicines and inability to make patients adhere to the services given by the facilities. The consequences of these factors can be dangerous to the patients as well as to the wider public and hence making available the appropriate human resource and HIV/AIDS related commodities including medicines should be the priority for the health facilities and the region to improve the quality of HIV/AIDS services in the study area.

## Introduction

Achieving accessible, quality healthcare for persons with HIV and AIDS is a critical need for patients living worldwide. Universal access to comprehensive health services in these patients substantially reduce HIV related morbidity and mortality. These services play significant role in address issues related to counseling on HIV infection and its treatment, Prevention of HIV transmission, laboratory service, prophylaxis and treatment of opportunistic infections, Palliative care and integrating nutritional services throughout the continuum of HIV/AIDS care.^1^ Unfortunately, the health sector in many of the places in the world most affected by the disease is weak, and health services are faced with the shortages of human and financial resources. The scarcely available health professionals are also constrained by inadequate knowledge about HIV/AIDS patient care and lack of counseling skills especially for pediatrics groups. This is clearly demonstrated in sub-Saharan Africa, where people with HIV/AIDS-related illness occupy more than 50% of hospital beds, and where organizations and facilities providing care are overwhelmed by the demand.^2^,^3^ Many developing countries are unable to supply enough of the commodities necessary for the proper management of HIV/AIDS including laboratory equipments and antiretroviral medicines.^4^–^6^

Despite issuing national HIV/AIDS policy, establishing National AIDS Council, National AIDS Secretariat, and other relevant bodies to tackle multi-sectorial effects of HIV/AIDS, Ethiopia continued facing a mixed epidemic of this infection amongst the sub-populations and geographic areas. The disease caused a significant decrease in life expectancy and a greatly reduced workforce. A number of children became orphaned and many aged population left with no care.^7^ Evidences indicated that there is a need for robust HIV/AIDS care and support services in Ethiopia, including treatment of opportunistic infections, nutritional support, palliative care and pain management to HIV/AIDS patients.^8^ Most, if not all, health facilities are overcrowded with patients suffering from a variety of infectious diseases. All health facilities built prior to 2005 never considered the structures that HIV & AIDS programs require. Private sections for counseling and testing are needed. Internal facilities, like simple furniture, are close to non-existent, or are in short supply almost everywhere. The training of health workers at all levels was in adequate to address the HIV & AIDS issues. There are still too few health providers in Ethiopia and these providers are crowded with a high load of patients and hence were unable to provide expected services to their patients.

The scarcity of human resource along with insufficient laboratory supplies made laboratory service and medical record handling poor and resulted in poor patient retention in health facilities.^9^,^10^ Ensuring adherence to ART regimens remains major challenge of the ART program in the country and little has been done to evaluate the quality of the services delivered.^11^,^12^ Therefore, it was found important to undertake this services quality assessment so as to help in identification of problem areas and to generate ideas for resolving the identified problems.

## Methods

### Study context

Facility based descriptive cross-sectional study was conducted from February to May, 2017 in selected HIV/AIDS services' providing health centers of East Shoa Zone, Oromia Regional State. Excluding those under construction, the zone had a total of 356 public health facilities (4 hospitals, 60 health centers and 292 health posts) of which 18 (4 hospitals and 14 health centers) were giving HIV/AIDS services to a total of 16154 HIV/AIDS patients in the zone. Public hospitals were serving 9943 patients while the remaining 6211 patients were served at the 14 health centers.^13^

### Sampling technique and sample size determination Health facility selection

The sample of health facilities required for this study was determined using Logistic Indicators Assessment Tool (LIAT) prepared by USAID/DELIVER PROJECT. This document suggested that at least 15% of the target health facilities should be selected as a sample for conducting such study.^14^ Accordingly, 57% (8 of the total 14 health centers) were selected as a study sample. The selection of the sample facilities was conducted considering the patient load of the health centers. Extreme/deviant sampling technique was utilized to include the 8 health centers (4 with high patient load and 4 with low patient load) in the study.

### Selection of health professionals

ART coordinator, MCH coordinator, Laboratory representative, TB focal person and store managers from each of the selected health centers participated in the survey.

### Selection of Medicines

A total of 11 ARV medicines, 10 anti-TB medicines, 5 anti-pain medicines and 4 opportunistic infection medicines were selected and assessed. The selection of ARV medicines was made based on the national guideline of comprehensive HIV/AIDS prevention, care and treatment^15^ and the selection of anti-TB medicines was made based on national TB management guideline.^16^ Anti-pain medicines and other opportunistic infections medicines were selected based on Standard Treatment Guideline for health centers^17^ and most commonly used medicines at the health centers.

### Data collection methods and instruments

The selected health professionals were interviewed to assess the types of services given using researcher administered structured questionnaires that were prepared based on USAID Health care improvement project tools18 and translated to the local language (i.e to Afan Oromo). The availability of HIV/AIDS related medicines were checked using Logistics Indicators Assessment Tool prepared by USAID14 and store managers interviews.

Observation checklist was used to check the availability of laboratory supplies and equipments and different HIV/AIDS services related guidelines/documents. Data was entirely collected by the principal investigator.

### Data quality assurance

Appropriately designed data collection methods and instruments were used. The instruments were pretested on two health centers and necessary modification was made before starting the actual study. Every day the collected data was reviewed and checked for completeness and consistency of the response.

### Data entry and analysis

The collected data was checked for consistency and completeness of information and analyzed using SPSS version 20.

## Results

### Types of services given to HIV/AIDS patients TB diagnosis and treatment for HIV positive patients

All health centers were offering anti-TB services including counseling on TB and anti-TB medicines, screening for active TB, Isoniazid preventive therapy and treatment of active TB (DOTS) to HIV positive patients. Two of the eight health centers (25.00%) were also providing treatment of MDR TB but the remaining six health centers were not offering the service because of scarcity of human resource and hence MDR TB patients were referred to nearby facilities. All health centers had a separate room for anti-TB service provision. The respondents said that nurses were the main providers for the majority of anti-TB services even though other professionals like laboratory specialists, health officers and druggists were involved in diagnosing the infection, prescribing medicines and in providing medicine counseling respectively. TB services guideline was available and was reported to be used as needed in 7 (87.50%) health centers. TB services coordinator from the remaining health center said that the guideline was lost because of aging and poor handling.

### Family planning services integrated with HIV/AIDS care

Respondents from all of the visited facilities said that their health centers were offering family planning services including counseling on family planning methods available and methods' ability to prevent sexually transmitted infections and HIV/AIDS (i.e condom). Long acting family planning methods like implants, injection contraceptive (Depo-provera) and IUCD (Intra Uterine Contraceptive Device) were provided in a room prepared for family planning service provision while condoms and oral contraceptive pills were given both at the ART pharmacy and family planning room. These services were provided by midwifes, nurses and druggists. Five health centers (62.50%) had family planning guideline at their facilities but only 2(40.00%) of them referred to it while delivering the services.

### Home based care provision to HIV/AIDS patients

Only two of the eight health centers were providing home based care to HIV/AIDS patients. Respondents from these facilities said that the care targeted those patients whom the disease harmed to a higher degree (stage three and above) and those who were economically weak. These two facilities were providing home based care in collaboration with organizations named OSSA (Organization of Social Service Agents) and WFP (World Food Program). Health care teams from these facilities, Volunteer community members and delegates from the two organizations had usually visited patients' home and provided psycho social and economic support. The care providers offered information to patients and their family members on how to manage common health problems at home, infection prevention and control and proper nutrition. Basic supplies including soap, oil and food powder contributed by these organizations were also delivered to the patients by the home-based care provider team. The health centers on their side arranged transportation, free phone call to the colleagues and patients and room for discussion to the care team as needed.

### Psycho-social and nutritional support to HIV/AIDS patients

Support groups onsite (health professionals), patient support groups, support groups composed of volunteer community members and NGO based groups were the major support groups established to help patients cope with psychological, economic and social consequences of the infection. Respondents from all health centers said that they had one or more of these support groups at their facility. The respondents stated that, despite their existence, the first three support groups have never done well when compared to the available NGOs and against the plan they were established for. Business of the support groups with other activities and lack of motivation were stated to be the main reasons. NGOs named New Generation Support Group, OSSA and Hunde were counseling patients against social stigma and discrimination and supported them by food and finance in some of the studied health centers. Respondent from one health center said that Catholic and protestant churches available in the area were supporting pediatric HIV positive students through covering foods, school fees, uniforms and other learning materials.

Only three facilities had a system to follow whether patients were in support group. Respondents from these facilities said that their health centers checked the membership and stay of patients in support group by asking the patients and by checking with the support groups.

### Pre-ART and ART services

Pre-ART and ART services have been offered at the study facilities for 4 to 12 years (Mean =7.88, SD=2.36). ART coordinators from all study facilities said that their facilities were providing HIV/AIDS counseling, partner counseling (if in couple), cotrimoxazole prophylaxis, WHO disease staging, weight measurement and opportunistic infections treatment to HIV/AIDS patients. ART and anti-pain medicines prescription and dispensing and medicine adherence counseling and assessment were also given at all health centers. The respondents said these services were provided by health officers, druggists, pharmacists, and adherence counselors available at the facilities. Three health centers had both pharmacists and druggists providing ART services while the remaining provided the services with either pharmacists only (two health centers) or druggists only (three health centers). ART service guideline and HCT guideline were available in all facilities although they were rarely used in practice.

ART coordinators from all health centers said that patients' appointment schedule for ART services (which was mostly every two weeks, monthly or bimonthly) was based on whether the patient was new or repeated, availability of ART medicines, adherence history and distance the patient had to travel to the facility. Patients who were new and resided closer to the health center were initially appointed every two weeks in all health centers. This was done mainly to check their adherence status to the prescribed medication and the side effects/adverse effects resulting from medicines. Patients coming from distance and chronic patients were frequently appointed monthly or bimonthly even though they were sometimes appointed for two weeks in case ART medicines were not adequately available. ART coordinators in all facilities stated that despite the schedule, not all patients respect their appointment date. They stated that a number of patients missed their appointment date mainly due to distance from facility, forgetting the appointment date, side effects of medicines, economic problem (lack of money for transport) and fear of stigma and discrimination from peers and society. They also said that some patients missed their appointment because of inconvenience due to their occupation (government/private employee, daily laborer, house maid), believing that they get cured by holy water, overlap with social issues (idir, mahber, death of relatives/neighbours) and thinking that the first dose has cured the disease.

### Prevention of Mother To Child Transmission (PMTCT) services

Prevention of mother to child transmission services were available in all the surveyed facilities. All facilities were providing HIV/AIDS counseling, mother and infant prophylaxis at delivery and enrollment of mothers and infants in HIV care program. MCH coordinators from six health centers said that they had professionals trained in PMTCT services including health officers, nurses, midwives and druggists.

### HIV/AIDS laboratory services

There were a total of 17 laboratory specialists in the study facilities of which 5 had first degree and 12 had diploma in medical laboratory. These laboratory specialists were providing HIV testing and other HIV/AIDS related services in the study health centers. Even though the laboratory specialists rarely used it in practice, majority, 6 (75.00%) health centers had written laboratory protocols with set of standards for conducting HIV test. National protocols for test disclosure and Material Safety Data Sheet (MSDS) for chemicals used and stored in laboratory were available in 5 (62.50%) and 3 (37.50%) health centers respectively. Only three laboratories (37.50%) had chemical containers clearly labeled with identity, hazard warning and date. Some of the remaining laboratories had unlabeled chemical containers and some others had scratched and/or dim labeling. Laboratory room, supplies and equipments were observed being hygienic and healthy in 4 (50.00%) health cnters. Protective gloves and gowns were available and in use in all the visited laboratories while nose masks were available and in use only in 4 (50.00%) laboratories. Of the eight health centers only two facilities had fire extinguisher in the laboratory while none of the facilities had eye protection for the laboratory service providers.

### Availability of ARV and related medicines

Majority of the study facilities, 6 (75.00%) were using only bincards for controlling the stock movement of ARV and other medicines while two facilities (25.00%) were using both bincard and electronic system (HCMIS). All health centers lacked bincard for one or more of ARV, OI and anti-pain medicines. The mean number of anti-TB, ARV and antipain medicines that had bincard at the time of visit was 8.13, 7.13 and 3.12 respectively. On average 81.25% of anti-TB medicines and 64.77% of ARV medicines had bincard at the time of visit. The average percentage of anti-TB medicines that had updated bincard was 74.97% while 57.29% antipain medicines had the same (Table 1).

**Table 1:** Availability and update status of bincards for ARV medicines, anti-TB and other opportunistic infection medicines in health centers of East Shoa Zone, Oromia, Ethiopia, 2017

	Bincard available		Bincard updated	
	
	Mean(SD)	(Min,Max)	Mean(SD)	(Min,Max)
No.ARV medicines	7.13(1.72)	(4,9)	3.75(2.05)	(1,7)
%ARV medicines	64.77(15.70)	(36.35,81.82)	49.46(18.20)	(25.00,77.78)
No.Anti-TB medicines	8.13(0.99)	(7,10)	6.12(2.03)	(4,10)
%Anti-TB medicines	81.25(9.91)	(70.00,100.00)	74.97(19.90)	(44.40,100)
No.OI medicines other than anti-TB	2.38(0.74)	(2,4)	1.25(0.46)	(1,2)
%OI drugs other than antiTB	59.38(18.60)	(50.00,100.00)	52.08(5.89)	(50.00,66.67)
No.Anti-pain medicines	3.12(0.64)	(2,4)	1.87(1.36)	(0,4)
%Anti-pain medicines	62.5(12.81)	(40.00,80.00)	57.29(34.92)	(0.00,100.00)

OI= Opportunistic Infection, No = Number

Over all 6 (75.00%) of the health centers faced the stockout of one or more ARV medicines on the day of visit while all health centers, 8 (100.00%) reported they had stockout of one or more ARV medicines in six months prior to the study. The stockout was high for NVP 240ml (50mg/5ml) syrup, 4 (66.70%) and AZT300/3TC150/NVP200, 5 (62.50%) on day of visit and in the past six months respectively. Regarding anti-TB medicines, four facilities did not experience stockout on day of visit on all anti-TB medicines studied while three had the stockout of E100. Five health centers (62.50%) stated that they had the stockout of one or more anti-TB medicines in the six months prior to the study. The highest stockout was for INH100, 4 (80.00%) and the lowest was for E100, 2 (40.00%). Six health centers (75.00%) experienced the stockout of one or more opportunistic infection medicines (other than anti TB medicines) on the day of visit and in the six months prior to the study. Cotrimoxazole 960mg tablet was the OI medicine with the highest stockout on day of visit, 4 (66.70%) as well as in the six months prior to the study, 5 (83.30%). Six (75.00%) and 7 (87.50%) of the study facilities experienced the stockout of one or more commonly used anti-pain medicines on the day of visit and in six months prior to the study respectively. Considerable number of study facilities, 4 (66.70%) had the stockout of tramadol 50mg tablet on day of visit and ibuprofen 400mg tablet in six months prior to the study, 5 (71.40%). None of the study facilities faced the stockout of paracetamol 500mg tablet and diclofenac 100mg tablet both on day of visit and in six months prior to the visit (Table 2).

**Table 2:** ARV, Anti-TB, OI and anti-pain medicines stockout on the day of visit and within six months prior to the study in health centers of East Shoa Zone, Oromia, Ethiopia, 2017

	Drug type	HCs Stockedout of each medicine on day of visit, N(%)	HCs Stockedout of each medicine within six months prior to the study, N(%)
ARV medicines	TDF300/3TC300/EFV600	0(0.00)	3(37.50)
	AZT300/3TC150/NVP200	0(0.00)	5(62.50)
	AZT300/3TC150	2(33.30%)	2(25.00)
	TDF300/3TC300	0(0.00)	0(0.00)
	AZT60/3TC30/NVP50	0(0.00)	1(12.50)
	AZT60/3TC30	1(16.70)	0(0.00)
	EFV600	0(0.00)	1(12.50)
	NVP200	1(16.70)	2(25.00)
	EFV200	0(0.00)	3(37.50)
	EFV50	0(0.00)	0(0.00)
	NVP240ml syrup(50mg/5ml)	4(66.70)	2(25.00)

Anti TB and other OI medicines	RHZE150/75/400/275	0(0.00)	0(0.00)
	RH150/75	0(0.00)	0(0.00)
	RHZ60/30/150	0(0.00)	0(0.00)
	RH60/30	0(0.00)	0(0.00)
	RH60/60	0(0.00)	0(0.00)
	E400	0(0.00)	0(0.00)
	1NH300	0(0.00)	3(60.00)
	Streptomycin vial 1gm	0(0.00)	0(0.00)
	E100	3(75.00)	2(40.00)
	INH100	1(25.00)	4(80.00)
	Cotrimoxazole 240/5ml syrup	0(0.00)	3(50.00)
	Cotrimoxazole 480mg tab	3(50.00)	4(66.70)
	Cotrimoxazole 960 mg tab	4(66.70)	5(83.30)
	Ciprofloxacin 500mg tab	2(33.30)	3(50.00)

Anti-pain medicines	Paracetamol 120mg/5ml syrup	3(50.00)	4(57.10)
	Paracetamol 500mg tab.	0(0.00)	0(0.00)
	Ibuprofen 400mg tab.	2(33.30)	5(71.40)
	Diclofenac 100mg tab.	0(0.00)	0(0.00)
	Tramadol 50mg tab.	4(66.70)	4(57.10)

The mean number of stockedout OIs medicines (excluding anti-TB medicines) on day of visit was 1.50 while the mean percentage stockout for these medicines on the same day was 37.50%. OIs medicines (excluding anti-TB medicines) and anti-pain medicines respectively had a mean percentage stouckout of 53.57% and 32.50% in six months period prior to the study (Table 3).

**Table 3:** Mean ARV, Anti-TB, OI and anti-pain medicines stockout on day of visit and within six months prior to the study in health centers of East Shoa Zone, Oromia, Ethiopia, 2017

	On day of visit		In the past six months	
	
	Mean±SD	(Min,Max)	Mean±SD	(Min,Max)
No.of ARV medicines stockedout	1.33±0.52	(1,2)	2.50±1.41	(1,5)
% of ARV medicines stockedout	12.12±4.69	(9.09,18.08)	22.72.±12.85	(9.09,45.45)
No.of anti-TB medicine stockedout	1.00±0.00	(1,1)	1.80±0.84	(1,3)
% of anti-TB medicines stockedout	10.00±0.00	(10,10)	18.00±8.37	(10,30)
No.of OI medicines stocked out	1.50±0.55	(1,2)	2.14±1.21	(1,4)
% of OI medicines stockedout	37.50±13.69	(25,50)	53.57±30.37	(25,100)
No.of anti-pain medicines stockedout	1.29±0.76	(1,3)	1.63±0.74	(1,3)
% of anti-pain medicines stockedout	25.71±15.12	(20,60)	32.50±14.88	(20,60)

No = Number

**Figure 1:**
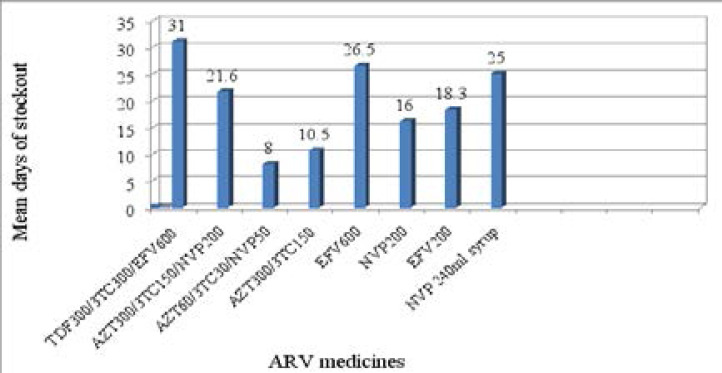
Mean stockout days of ARV medicines within six months prior to the study in health Centers of East Shoa Zone, Oromia, Ethiopia, 2017

TDF300/3TC300/EFV600 was the ARV medicine with the longest stockout duration in six months period prior to the study (Mean=31.00, SD=10.54 days). EFV 600 (Mean=26.50, SD=13.43 days) and NVP240ml syrup (mean=25.00, SD=8.49 days) also had longer stockout duration while AZT60/3TC30/NVP50 (Mean=8.00 days) had the shortest stockout duration in six months period prior to the study (Figure 1). Failure to order on time and inability to receive the ordered quantity on time (due to shortage at the suppliers' store) were reported to be the main reasons for the stockout of the ARV medicines. Store managers said that their facilities have made emergency order and borrowed from nearby facilities to mitigate the stockout of these ARV medicines. They also stated that they were dispensing below the prescribed quantity in case they suspect drugs could stockout soon and there was uncertainty on timely supply.

Ciprofloxacin tablet (Mean=43.00, SD=7.55days) and Cotrimoxazole syrup (Mean=36.30, SD=15.01 days) had longer stockout duration compared to other OI medicines including anti-TB medicines. The mean stockout duration of INH100 in six months period prior to the study was 24.30 (SD=16.80) days while the remaining two stockedout anti-TB medicines, INH300 and E100 had mean stockout duration of 23.30 (SD=9.71) days and 22.50 (SD=17.68) days respectively. Among the commonly used anti-pain medicines, Ibuprofen tablet had a mean stockout duration of 22.60 (SD=8.23) days while paracetamol syrup had a mean stockout duration of 21.50 (SD=4.93) days (Table 4). Failure to order on time and inability to quantify in enough amount were mentioned as the main reasons for the stockout of OI and anti-pain medicines. The facilities reported that they were dispensing one anti-pain and OI medicines (not anti-TB medicines) instead of the other and were dispensing below the prescribed quantity (in case they suspect stockout in near future) to solve the problems of stockout. The facilities stated that they were borrowing anti-TB medicines from nearby facilities and were placing emergency order for mitigating the stockout of anti-TB medicines.

**Table 4:** Mean stockout days of Anti-TB and other OI medicines and most commonly used anti-pain medicines within six months prior to the study in health centers of East Shoa Zone, Oromia, Ethiopia, 2017

	Medicine type	Mean days of stockout (SD)
Anti-TB and other OIs medicines	INH300	23.30(9.71)
	INH100	24.30(16.80)
	E100	22.50(17.68)
	Cotrimoxazole 240mg/5ml syrup	36.30(15.01)
	Cotrimoxazole 480mg tab.	19.75(11.80)
	Cotrimoxazole 960mg tab.	22.50(10.54)
	Ciprofloxacin 500mg tab.	43.00(7.55)

Anti- pain medicines	Paracetamol 120mg/5ml syrup	21.50(4.93)
	Ibuprofen 400mg tab.	22.60(8.23)
	Tramadol 50mg tab.	18.50(9.61)

OIs=Opportunistic Infections

## Discussion

### Types of services delivered to HIV/AIDS patients

The present study revealed that all health centers were offering anti-TB services to HIV/AIDS patients including counseling on TB and anti-TB medicines, screening for active TB, isoniazid preventive therapy and treatment of active TB (DOTS) to HIV positive patients. This goes in line with WHO recommendation regarding the package of care that should be given to HIV/AIDS patients so as to tackle the health and economic consequences of this infection.^19^ All health centers in this study had a separate room for providing anti-TB services similar to a study from Malawi.^18^ Provision of these services in a separate room prevents the contamination of healthy person by the diseased and avoids the spread of this infection.

The current study indicated that family planning methods (with counseling) including condoms, oral contraceptive pills, injectables, implants and IUCD were available in all the study facilities and hence patients had an opportunity to choose contraceptive method they prefer. This contradicts a study conducted in Nigeria that showed up to 35% of female clients on ART had unmet contraceptive needs.^20^

As per this study, some health centers had no family planning guide line and there was also lower/zero utilization rate in the facilities where the guideline was available. This may compromise the quality of the services delivered and hence the availability of the guideline and its utilization rate needs to be improved.

Home based care has the advantage of offering an adjustable and flexible care provision for HIV/AIDS patients as well as their affected families.^21^ This program largely supplements the formal health care provided at health facilities through providing psychosocial, economic, palliative and nutritional supports at home. Despite its high significance however, such care rarely exists in low income countries including Ethiopia.^22^,^23^ This parallels the finding of this study which indicated only two of the eight health centers (25%) were providing home based care. All facilities in the current study had support group/s established to help patients cope with psychological, economic and social consequences of the infection similar to the findings of the study conducted in Malawi18 but these support groups were found to be performing below what was expected from them. The low performance happened due to workload of the support groups and lack of motivation.

The current study documented that all the study facilities were providing HIV/AIDS counseling, partner counseling, cotrimoxazole prophylaxis, WHO disease staging, weight measurement and prevention/treatment of opportunistic infections to HIV/AIDS patients. They were also offering ART and anti-pain medicines prescription and dispensing and medicine adherence counseling and assessment to HIV/AIDS patients as per WHO ART guideline and the Ethiopian HIV prevention, care and treatment guideline.^15^,^24^

Regarding the availability of ART service guideline and HCT guideline, it was found that all the studied facilities had ART and HCT guideline but they rarely used them in practice. The lower utilization rate of these documents might have resulted from the service providers' assumption that they knew the contents of the documents and hence they wouldn't need to refer to them. This lower utilization rate may reduce the quality of the services delivered and therefore need to be improved.

Even though patients in the current study were frequently appointed to receive their ART services biweekly, monthly or bimonthly similar to a study from Addis Ababa^25^, the respondents said that a number of patients missed their appointment dates. This happened mainly due to distance from facility, forgetting the appointment date, side effects of drugs, economic problem (lack of money for transport) and fear of stigma and discrimination from peers and society. Such non-adherence to schedule hinders the ART program and opens door for disease progression, development of drug resistance and death.^26^

The current study indicated that all of the studied health centers were providing PMTCT services including HIV/AIDS counseling, mother and infant prophylaxis at delivery and enrollment of mothers and infants in HIV care program. This agrees with WHO as well as Ethiopian PMTCT guidelines that recommended the provision of PMTCT services as one of the main strategies towards eliminating HIV infection.^19^,^27^ It also agrees with facility-based studies done in Ethiopia that indicated PMTCT has been expanded in accelerated fashion throughout the country currently with most public hospitals and health centers providing these services.^28^ This study also revealed that PMTCT service providers in majority of the health centers (87.50%) had taken in service training and this agrees with WHO guideline and Ethiopian HIV prevention, care and treatment guideline which recommended training of health workers to ensure high quality care and timely implementation of updated national policies.^15^,^24^

Most health centers in the current study had written protocols for conducting HIV test even though they were said to be rarely used in practice. The availability of material safety data sheet and fire extinguisher were minimal. Mboera et al found similar result in a study they conducted in Tanzania addressing the readiness of the national health laboratory system in supporting care and treatment of HIV/AIDS.^29^

The present study identified labeling problem on chemical containers in majority of the health centers. Chemical containers in these facilities were either unlabeled or lacked clear labeling for identity, hazard warning and date and this may lead to difficulty in identifying chemicals and hence wrong use of one chemical instead of the other. Lack of clearly visible expiry date on the containers may cause use of expired chemical. Laboratory room, supplies and equipments were observed to be unhygienic in 50% health centers and this may create favorable environment for microorganisms that can put health of the patients and service providers at danger and may degrade patient satisfaction with the services delivered.

### Availability of ARV and related medicines

Findings from the current study indicated that majority of the health centers, 6 (75%) faced the stock out of one or more ARV medicines on the day of visit and all health centers, 8 (100%) reported the stock out of one or more ARV medicines in six months prior to the study. This dis agrees with a study done in Oromia National Regional State that has shown the availability of ARV medicines in health centers of the region to be 100%.^30^ It also disagrees with the study done in Ethiopia that showed the stock out to be non-existent or minimal for ARV medicines.^31^ The stockout in the current study may imply that the suppliers need to improve their pharmaceutical management system (to overcome medicines shortage in the store which was stated as one of the causes of medicines stockout in health facilities) and the health centers should see the quantity they order and when they order. The number of facilities that experienced the stockout of ARV drugs was much higher than the national survey conducted in South Africa, where 14% of the facilities reported a stock out of adult ARV drugs and 6% for pediatric ARV drugs.^32^ The higher stockout in the current study might be related to the difference in the level of development between the two countries that could influence the supply of pharmaceuticals to health facilities and the differences in the structure of supply chain management between the two countries.

Pasquet et al stated that stockout of ARV medicines (either due to facility problem and/or supplier problem) put patients at risk of disease progression and death, in drug resistance development, hampers progress towards universal access, and diminishes the credibility of ART programs in the eyes of patients, community and healthcare providers and generally put the public health in danger.^26^ This evidence indicates that the study facilities and the suppliers are expected to work towards solving the problem of ARV medicines stockout, mainly those with relatively longer stockout duration (TDF300/3TC300/EFV600, EFV600, NVP240ml syrup).

Continuous availability of proper anti-TB medicines is a prerequisite to ensure the correct clinical management of TB and to reduce health care cost associated with it.^33^ Despite this however, only half of the study facilities had all the required anti-TB medicines on day of visit in the current study. Five health centers (62.50%) also reported that they faced the stockout of one or more anti-TB medicines in the six months prior to the study. Such an interrupted availability of medicines in the facilities put patients at risk of treatment failure/drug resistance^33^ and hence needs to be addressed properly.

Prevention and treatment of opportunistic infections other than TB is an essential component of comprehensive HIV/AIDS treatment and care. However, certain health facilities in the developed and developing world do not have access to medicines required to prevent/treat these infections and hence many patients have OIs.^34^ The present investigation also showed that majority of the health centers (75.00%) had shortage of one or more of these OIs medicines on the day of visit and in the six months prior to the study. This shortage put patients' health at risk and degrades their trust in health system26 and hence should get a solution.

The present finding indicated that more than 70% of the study facilities experienced the stockout of one or more commonly used anti-pain medicines on the day of visit as well as in six months prior to the study. Congruent to this finding, a study conducted in selected facilities on all regions of Ethiopia indicated stock out was present for almost all kinds of pain-relieving medicines.^35^ Stock out was more often reported in the above study for Ibuprofen and tramadol tablets compared to the other analgesics just similar to the current study where considerable number of study facilities (71.40% for Ibuprofen and 66.70% for tramadol) experienced the stockout of these medicines.^35^ Findings from the current study is also supported by the World Health Organization report that estimated 5 billion people living in countries with no or insufficient access to treatment for pain. In these countries, nearly 1 million HIV/AIDS patients are suffering without adquate pain treatment each year.^36^

## Conclusion

The study facilities were providing different services to HIV/AIDS patients including continuum of care services, ART services, PMTCT services and HIV/AIDS laboratory services. They faced different challenges while delivering these services including scarcity of human power and failure to make patients adhere to the services properly. Majority of the study facilities faced the stockout of NVP 240ml syrup and AZT300/3TC150/NVP200 from ARV medicines and they experienced the stockout of INH 100 and E 100 from anti-TB medicines. Cotrimoxazole 960 mg tablet, Ibuprofen 400mg tablet and Tramadol 50mg tablet were also the main medicines whose stockout was reported.

## Recommendations

Health facilities should hire additional human power so as to improve the quality of the services given to patients, especially anti-TB services and home-based care. Health centers should quantify medicines properly and order them on time and suppliers should store enough of these medicines and supply them to facilities as requested. Concerted effort aimed at strengthening patient adherence services is needed. Additionally, Service providers should properly use the available service guidelines and facilities should communicate zonal health office and other concerned body for unavailable guidelines. Laboratory room, supplies and equipments should also be kept hygienic and chemical containers in the laboratory should be properly labeled.

## Acronyms

ACORD-Agency for Co-operation and Research in Development.

AIDS- Acquired Immune Deficiency Syndrome.

ART-Anti Retroviral Therapy.

ARV-Anti Retro Viral.

EPHA-Ethiopian Public Health Association.

EPHI-Ethiopian Public Health Institute.

FHAPCO-Federal HIV/AIDS Prevention and Control Office.

FMHACA-Food, Medicine and Health care Administration and Control Authority.

FMOH-Federal Ministry Of Health.

GHTAP-Global Health Technical Assistant Project.

HCMIS-Health commodities Management Information System.

HIV-Human Immune deficiency Virus.

HCT-HIV/AIDS Counseling and Testing.

ILO-International Labor Organization.

LIAT-Logistics Indicators Assessment Tool.

MCH-Maternal and Child Health.

MDR TB-Multi Drug Resistant Tuberculosis.

MSH-Management Science for Health.

NGO-Non-Governmental Organization.

OI- Opportunistic infection.

PMTCT- Prevention of Mother To Child Transmission.

SD-Standard Deviation.

SPSS-Statistical Package for Social Sciences.

WHO-World Health Organization.

USAID-United States Agency for International Development.
